# Who comes when the world goes Code Blue? A novel method of exploring job advertisements for COVID‐19 in health care

**DOI:** 10.1002/nop2.721

**Published:** 2020-11-30

**Authors:** Rory D. Watts, Devin C. Bowles, Colleen Fisher, Ian W. Li

**Affiliations:** ^1^ School of Population and Global Health The University of Western Australia Perth WA Australia; ^2^ Australian National University Canberra ACT Australia; ^3^ Council of Academic Public Health Institutions Australasia Canberra ACT Australia

**Keywords:** job advertisements, nurses, nursing, supply and demand, workforce

## Abstract

**Aim:**

To explore the health workforce responses to COVID‐19.

**Design:**

Analysis of job advertisements.

**Methods:**

We collected advertisements for healthcare jobs which were caused by and in response to COVID‐19 between 4 March–17 April 2020 for the United States, Canada, United Kingdom, Australia and New Zealand. We collected information on the date of the advertisement, position advertised and location. We categorized job positions into three categories: frontline, coordination and decision support.

**Results:**

We found 952 job advertisements, 72% of which were from the United States. There was a lag period between reported COVID‐19‐confirmed cases and job advertisements by several weeks. Nurses were the most advertised position in every country. Frontline workers were substantially more demanded than coordination or decision‐support roles. Job advertisements are a novel data source which leverages a readily available information about how workforces respond to a pandemic. The initial phases of the response emphasise the importance of frontline workers, especially nurses.

## 1 INTRODUCTION

Public health crises have a disproportionate effect on frontline healthcare workers and, in particular, nurses. Such crises not only affect healthcare workers in terms of workload and potential risks; they also exacerbate existing economic problems, where the distribution of limited workers has a drastic effect on the outcomes of patients. The COVID‐19 crisis is not different and the allocation of workers in response to this crisis is critical.

During such crises, efforts should be made to understand the responses of healthcare providers and healthcare systems. The reason for this is twofold, as it allows unaffected countries to learn from countries who have already experienced the early phases of the crisis and it allows decision‐makers to mitigate future crises by drawing from an evidence base. Job advertisements are one such method which represents a readily accessible data source to explore the speed, magnitude and composition of pandemic responses. Despite this, analysis of job advertisements is under‐used, relative to the benefit they can provide.

### 1.1 Background

COVID‐19 has changed health care and society. By the end of June 2020, there were over 10 million confirmed cases and 500,000 deaths worldwide (Roser et al., 2020). The pandemic has drastically affected livelihoods, with economic slowdown and unemployment in many countries. At the centre of the crisis are healthcare workers and nurses in particular many of whom have increased workloads and increased working risks. Early research shows a relationship between COVID‐19 work and stress, self‐rated anxiety (Huang et al., 2020) and other mental health phenomena (Liu et al., 2020). With these findings is knowledge that this may only be the beginning of the crisis, with fears of a second wave in many countries, owing to the low rate of background immunity (City of Boston, 2020; Ministry of Health & Spain, 2020; New York State, 2020; Public Health Agency of Sweden, 2020; Public Health England, 2020; Wu et al., 2020) and the easing of restrictions.

It is important to conduct research about the response to COVID‐19 to mitigate the current pandemic and to prepare for similar events in the future. Pandemic preparedness literature tends to focus on evidence‐based clinical decision‐making, but evidence‐based decision making outside this area is less common. One aspect of pandemic preparedness that can be aided through evidence is workforce planning, especially for health workforces and frontline workers, such as nurses. One aspect of workforce research in this area is surge capacity and whilst surge capacity research is an important aspect of pandemic preparedness, such research is often focused on scenarios which are acute and localized (Einav et al., 2014; Traub et al., 2007), neither of which are properties of COVID‐19. It is also beneficial for planners to observe how countries are responding at a systems level, particularly among countries where intelligence sharing is well established. The “Five Eyes” countries of United States, United Kingdom, Canada, New Zealand and Australia are one such example.

Job advertisements are one data source which has the potential to inform workforce planning for pandemic preparedness, as advertisements provide a sense of how health systems are responding to demand and how demand is changing. Moreover, during a pandemic, when more than one profession or set of skills is being demanded, job advertisements may also provide a useful point of analysis beyond what could be captured by the review of only one profession, such as doctors or nurses. Job advertisements as a data source are flexible and have previously been used to examine skills that are required of individuals (Bennett, 2002), to examine trends in skills that are required in particular jobs (Todd et al., 1995) and to explore the demand for certain workforces over time (Watts et al., 2019). Using job advertisements to explore how workforces respond to a pandemic is novel and the use of readily accessible, time sensitive data, available in multiple countries, makes for a potentially rich contribution to workforce planning research, by providing a better understanding of workforce responses and health system stress.

## 2 THE STUDY

### 2.1 Aims

The aim of the study was to explore job advertisements for healthcare positions caused by and in response to COVID‐19, in terms of the number and location of advertised jobs, numbers of advertised jobs relative to confirmed COVID‐19 cases and positions advertised.

### 2.2 Design

We conducted an analysis of COVID‐19 job advertisements, defining a “COVID‐19” job advertisement as an advertisement where the advertised position was caused by and in response to, SARS‐CoV‐2. This means there was evidence that the job advertisement had arisen due to COVID‐19 and the position was aiding the response to it. In addition to this, we only considered health jobs and jobs which were specifically supporting healthcare responses such as cleaners or administrators.

### 2.3 Sample/Participants

We collected job advertisements through the online job board Indeed between 4 March–17 April 2020. We selected Indeed as it has country specific websites for each of the Five Eyes countries and Indeed is commonly used in those countries. For example, New Zealand currently has 14,000 job advertisements on Indeed (2.8 per 1,000 persons) and the United States has 2.6 m job advertisements (8 per 1,000 persons).

### 2.4 Data collection

We searched for COVID‐19 job advertisements by using the following search terms: “COVID,” “corona” and “pandemic.” Once we had collated these jobs, we filtered out false‐positive results through a combination of keyword searches and manual identification (true‐positive results were more likely to contain keywords such as emergency, response, COVID, urgent, etc.). We did not consider job advertisements which were exclusively in a language other than English (there were three Canadian advertisements exclusively in French which were excluded).

For job advertisements which fit our definition of a COVID‐19 job, we collected the following data: title of position (e.g. “COVID‐19 response urgent registered nurse”), job description, country and location advertised, the date the position was advertised and the date the information was collected. Locations of job advertisements were converted to geographic information through Google's GeoCoding API. In summary, the text description of a location (e.g. New York, NY) found in the job advertisement data was sent to Google, which processed the information and provided comprehensive, standardised information, including latitude, longitude and names of administrative regions (e.g. states, provinces).

The advertised positions (e.g. Registered Nurse, physical therapist) were categorised through iterative discussion with the research team. We categorised the advertised positions into roles (the above two examples would be categorised as “nursing” and “allied health,” respectively) initially and categorised those roles into broad positions within the COVID‐19 response: frontline, coordination and decision support (the above two examples would both be examples of “frontline” positions). The decision rules for categorisation are available in the Appendix S1.

### 2.5 Data analysis

Analysis was undertaken using Python version 3.7.4 in a Jupyter Lab environment. Handling of datasets was undertaken using Pandas and figures were generated using Plotly.

### 2.6 Validity, reliability and rigour

We compared our findings at a country level against confirmed cases of COVID‐19. For this, we used data published by Our World in Data (Roser et al., 2020), which publishes cases collected by the European Center for Disease Prevention and Control (European Centre for Disease Prevention & Control, 2020). For comparisons at the state level in the United States, we used data from the New York Times GitHub repository (New York Times, 2020). This repository compiles time series data from state and local governments and health departments. For both data sources, we used confirmed cases, defined as individuals whose coronavirus infections were confirmed by laboratory test and reported by a federal, state or local government agency. We chose to explore the state level response in the United States due to the large number of confirmed cases and job advertisements.

## 3 RESULTS/FINDINGS

**FIGURE 1:**
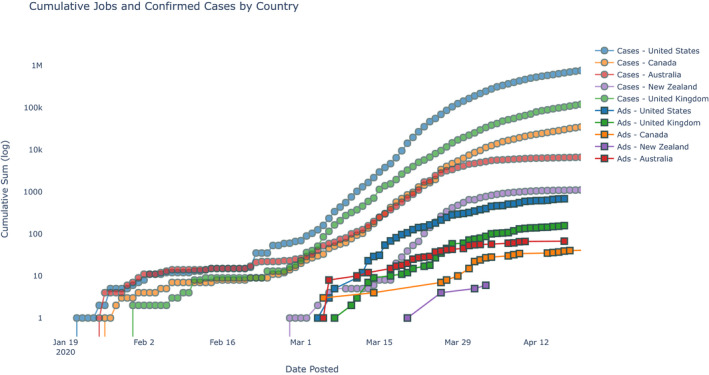
Cumulative jobs and confirmed cases by country

Figure 1 presents the cumulative sum of job advertisements per country alongside the cumulative sum of confirmed cases of COVID‐19 by date. Between 4 March–17 April 2020, there were a total of 952 advertisements for COVID‐19 jobs, 683 (72%) of which were from the United States, 157 (16%) from the United Kingdom, 67 (7%) from Australia, 39 (4%) from Canada and 6 (<1%) from New Zealand. These figures corresponded to a rate 1.02 advertisements per 1,000 confirmed cases in the USA, 1.52 in the UK, 1.30 in Canada, 10.31 in Australia and 5.52 in New Zealand.

In all countries, there was a lag between the growth of COVID‐19‐infected cases and growth of job advertisements. The lag between the first confirmed COVID‐19‐infected case and COVID‐19 job advertisement ranged from 21 days in New Zealand, to 43 days in the United States, with other countries having a similar lag to the United States. Furthermore, job advertisements in countries increased at a relatively consistent rate, with daily job postings becoming less frequent in April for Australia, Canada and New Zealand.

**FIGURE 2:**
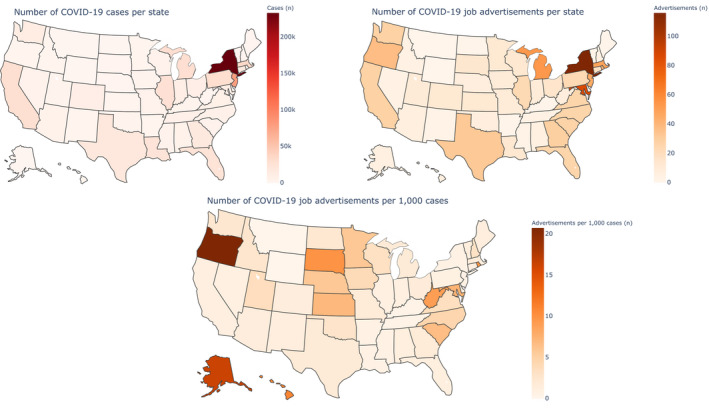
COVID‐19‐confirmed cases and job advertisements by State, United States of America

Figure 2 presents a series of heatmaps showing state level cases, advertisements and advertisements per 1,000 cases as of 17 April 2020 for the United States. The state of New York had the highest number of cases and the highest number of job advertisements in total. There were also high total numbers of cases and advertisements in New Jersey, Massachusetts, Texas and California. However, some states showed a much higher proportion of job advertisements relative to their reported cases (>10 job advertisements per 1,000 cases, where most states had 0.5–1.5 advertisements per 1,000 cases) including Oregon, Alaska, Hawaii and South Dakota.

Table 1 presents descriptive statistics for the positions advertised for COVID‐19. In their most aggregated form, 783 (86%) job advertisements were for frontline positions, 118 (12%) were for coordination positions and 14 (2%) were for decision‐support positions. For frontline positions, 293 (37%) advertisements were nursing positions, 98 (13%) were allied health positions and 57 (7%) were positions for medical doctors. Coordination positions commonly related to emergency planning (*N* = 50, 45%) and public health positions (*N* = 28, 25%). Decision‐support positions were mainly at universities, in positions including policy analysis, clinical trials, vaccine research or similar.

**TABLE 1:** Characteristics of advertised jobs

Parameter	Count (*N*)	% of total
Job advertisements	952	
United States	683	71.7
United Kingdom	157	16.5
Australia	67	7.0
Canada	39	4.1
New Zealand	6	<1%
Advertised position: frontline workers	783	82.2
Nurses	293	30.7
in United States	205	37.8^†^
in United Kingdom	38	26.6^†^
in Australia	37	58.7^†^
in Canada	12	46.2^†^
in New Zealand	1	20.0^†^
Allied health	98	10.3
Doctors	57	6.0
Advertised positions: coordination roles	118	12.4
Emergency planning	50	5.3
Public health	28	3.0
Advertised positions: decision support	14	1.5

^†^ Percentages of total jobs in respective country.

**FIGURE 3:**
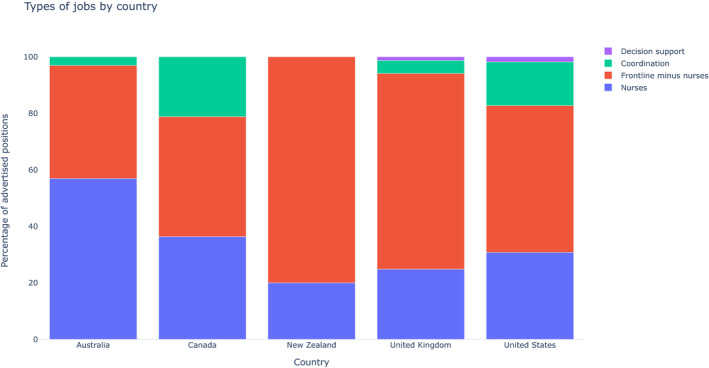
Types of job by country

Figure 3 presents the proportions of job types by country. For each country, the most common position advertised was nurses (between 20%–56% of all jobs) and frontline jobs were the most common advertised job category (between 78%–100% of jobs). All advertised nursing jobs were frontline jobs. There were much smaller proportions of positions advertised for coordination or decision‐support roles (0%–22% of jobs per country).

## 4 DISCUSSION

We explored COVID‐19 healthcare jobs in the United States, Australia, Canada, New Zealand and the United Kingdom, in March–April 2020. These jobs, as per our definition, are healthcare jobs which have been caused by and are in response to COVID‐19. However, it is expected that our findings are an underestimate, as similar positions may have been filled internally without advertisement, advertised outside Indeed, or existing positions may have been modified to compensate for additional tasks and duties. Therefore, our findings represent a subset of the workforce demand for COVID‐19 jobs.

The dynamics of job advertisements warrant discussion, particularly the lag between cases and advertisements and the consistent increase in jobs over time. Firstly, the lag between the rise in cases of COVID‐19 and the rise in job advertisements has multiple interpretations. Two likely interpretations are that existing personnel could handle the initial cases and that there was reluctance to advertise positions lest they were not needed. Another interpretation is that funding and approval for positions takes time, creating a delay between a surge in cases and a corresponding increase in advertisements. Secondly, a consistent increase in jobs is perhaps surprising given an exponential increase in confirmed cases, as a linear addition of frontline workers would soon be overwhelmed by cases. This may indicate that some hospital systems were yet to be overwhelmed, were internally reallocating workers or were constrained by budgets.

We found that US states differed in their rate of advertising COVID‐19‐related jobs. Whilst New York had the highest total number of cases and job advertisements, there were many states which had a much higher ratio of advertisements to cases. There are two reasons for this. Firstly, New York was one of the first states to report cases and, events in New York may have given other states good reason to prepare. Secondly, the virus has spread exponentially, and it is not clear that job advertisements could increase at the same rate, making any state with a large amount of cases have a much smaller ratio of advertisements to cases. Therefore, examining the ratio of advertisements to cases may only be sensible during the very early stages of a pandemic, or over a long enough time to capture corresponding job advertisement growth.

We provided a sense of scale of the advertised demand for different workforces during the initial response of a pandemic. There is a large demand for frontline workers, a substantially smaller demand for coordination positions and a smaller demand still for decision support. This may be a methodological artefact, for instance if coordination or decision‐support positions are more commonly internally reallocated than frontline workers. However, this finding is also unsurprising. Both coordination and decision‐support activities enjoy economies of scale well above the activities of frontline healthcare workers, especially during a pandemic with exponential spread. It will be useful to observe how demand for these workforces changes over time. For instance, one might expect a rise in the demand for researchers over time as more data becomes available.

Our findings also highlight the importance and vulnerability of nurses during a pandemic. In the countries we examined, nurses were the most advertised position and this is not surprising, considering that key components of a public health response (screening, supportive therapy, health education) rely on nursing duties and expertise (Nayna Schwerdtle et al., 2020). However, this implies that more nurses will bear greater risks of the pandemic and the anxieties which come with such risks if cases are not controlled (Shanafelt et al., 2020). The nature of COVID‐19 means that cases could overwhelm the supply of nurses and contingency plans should be considered. Initial suggestions have included expanding the pool of supply, such as students and retired nurses (Fraher et al., 2020) and might include the option to temporarily reallocate nurses across countries when overburdened.

Our research has demonstrated how insight can be generated using job advertisements as a data source. Job advertisements are a readily accessible data source which can provide information on the speed, magnitude and composition of a pandemic response. Further research would be welcomed, particularly research about methods to efficiently extract and compare information from job descriptions. For instance, we note that the content from our collected job descriptions was fifty pages long, much of which is irrelevant to understanding the role, tasks and duties of the position. Comparing job descriptions may not currently be an effective use of time, but new methods to extract key information could make this less time intensive and more valuable. It is also important to acknowledge that job advertisements may not be filled. This means that inferences about the roles, tasks and duties assume that the position is filled and that the filled role does not deviate substantially from the description. Related to this, research which periodically looks for re‐advertisements could be important, because researchers can ascertain a subset of positions which have not been filled which are highly demanded.

### 4.1 Limitations

We will not have captured all relevant job advertisements that fit our definition. Rather, we have captured a subset of the “true‐positive” job advertisements, where other true‐positive job advertisements may have been found using other job boards. However, we chose to use Indeed due to its ubiquity in the countries of interest. This, however, does not imply that similar groups in different countries use Indeed for the same purposes. For future investigations which focus on one country, an ensemble of job boards should be used, minimising the omission of key results. Furthermore, we were unable to identify “false negatives,” that is jobs which fit our definition but were not captured by our search criteria. This is because jobs may have been a response to COVID‐19 but may not mention this clearly in the job advertisement. It is also important to mention that there is no way to capture roles which have been created internally in an organisation, but which were not advertised publicly.

## 5 CONCLUSION

To the best of our knowledge, this is the first instance of job advertisements being used to monitor the response of workforces to a pandemic. Advertisements are an undervalued resource which can allow planners to look between countries and workforces, all in a relatively short amount of time.

In the context of COVID‐19, we have shown that job advertisements can be useful in exploring how workforces respond to the initial stages of a public health crisis, in terms of who is required, in what proportion and where. In the initial phases of the pandemic, there is an increased demand for frontline healthcare workers and of all workers; nurses are the most in demand. Considering that nursing care provides limited economies of scale when faced with an exponentially increasing disease, consideration must be given to the welfare of nurses and to exploring options if demand exceeds supply. Future research should investigate how the dynamics of workforce demand change over the course of the pandemic, and the potential use of other information contained in job advertisements, such as descriptions of tasks and duties.

## CONFLICT OF INTEREST

The authors declare they have no conflict of interest.

## AUTHOR CONTRIBUTIONS

RW, IL, DB, CF: Design. RW, IL, DB: Data collection. RW: Data analysis. RW: Writing. RW, IL, DB, CF: Drafting.

## ETHICAL APPROVAL

As our data were freely available in the public domain and no identifying features were collected from the data, we did not require ethics approval.

## PATIENT CONSENT

This paper does not involve patients; therefore, no patient consent is needed.

## Supporting information

Appendix S1

## Data Availability

The data used to inform this study are unavailable for public access at this time. Contact the corresponding author for further information.
