# Supplementary material for: Who comes when the world goes Code Blue? A novel method of exploring job advertisements for COVID‐19 in health care
Source: Nurs Open. 2020 Nov 30;8(3):1108–14. doi: 10.1002/nop2.721 (PMC9632666; doi:10.1002/nop2.721)
Supplement: Supplementary file 1 — Appendix S1 [file NOP2-8-1108-s001.docx]

Appendix S1

**Title: Categorisation rules for classifying job advertisements**

Our categorisation rules involved converting the names of advertised positions into roles, and then roles into broad categories (Frontline, Decision Support, Coordination).

We would recommend using this supplement to understand the categorisation process we used. However, we would not recommend implementing these rules in your own research, as the keywords listed, particularly for advertised positions were highly specific to our dataset. These keywords were a method to unambiguously categorise the positions we found and would likely produce errors if applied blindly to another dataset.

# Advertised Positions -> Roles

**'nurse'**: 'advanced practice', 'nursing', 'midwife', 'community response', 'nurses', 'nurse', 'rn',

'**nursing assistant'**: 'medication aide', 'patient care technician', 'dialysis technician', 'medication technician', 'nursing support', 'nursing assistant', 'emergency triage', 'cna',

**'doctor'**: 'locum', 'amsant', 'clinical director', 'gp', 'general practitioner', 'doctors', 'medicine', 'rmo', 'doctor', 'medical officer', 'physician', 'medical student'

**'allied health'**: 'social care roles', 'respiratory technician', 'dietary', 'counsellor', 'social care assessors', 'veterinarian', 'respiratory care', 'respiratory therapis', 'nutrition', 'therapist', 'physiotherapist', 'paramedic', 'respiratory trainee', 'respiratory therapy', 'x-ray', 'phlebotomist', 'phlebotomy', 'social workers', 'social work', 'imaging', 'radiographer', 'respiratory therapist', 'allied health', 'dietitian', 'pharmacy', 'pharmacist',

**'Not Specified / Multiple Roles'**: 'trust grade', 'ray technologists', 'resident assessment', 'radiology technician', 'radiologic tech', 'patient care tech', 'patient care associate', 'case manager', 'isolation', 'health recruitment', 'trainer consultant', 'general role', 'clinical professional', 'covid tech', 'surgical tech', 'emergency responder', 'emergency relief', 'emergency care', 'various healthcare', 'ward', 'x-ray', 'critical care', 'itu staff', 'response team', 'er tech', 'pool', 'bank', 'clinical temporary', 'clinical professionals', 'health care technician', 'immediate hires', 'medical technician', 'behavioral health', 'behavioural health', 'care aide', 'medical volunteer', 'general worker', 'care workers', 'medical assistant', 'monitor', 'medical provider', 'healthcare worker', 'support worker', 'caregiver', 'care assistant', 'caring partner', 'clinical support', 'community support', 'community',

**'administration'**: 'secretary', 'coronavirus coordination', 'concierge', 'administrative', 'admin', 'assistant', 'clerk',

**'support work'**: 'utility workers', 'shuttle driver', 'residential and supported living', 'receptionist', 'non-clinical', 'mask assembly', 'mechanic', 'interpreter', 'emergency shelter', 'non-clinical', 'recovery shelter', 'cityclean', 'nutritional', 'transport', 'disinfection technician', 'environmental services aide', 'dining', 'bank facilities management', 'web developer', 'accountant', 'food', 'domestic', 'porter', 'housekeeper', 'chef', 'armed response', 'cleaners', 'cleaner', 'cook', 'security'

**'non-specific phones/screening'**: 'test operative', 'remote emergency provider', 'drive thru', 'virtual covid', 'chat responder', 'screening', 'telephone', 'health advisor', 'screener', 'hotline', 'healthline', '111', 'call',

**'laboratory science'**: 'scientist', 'lab', ‘medical technologist', 'laboratory', 'lab technician',

**'coder/scribe'**: 'nhs it', 'medical writer', 'medical records', 'help desk', 'desktop', 'data validation', 'coder', 'scribe',

**'emergency planning'**: 'services coordinator', 'response director', 'hygiene manager', 'senior officer', 'emergency planner', 'material coordinator', 'support specialist', 'direct support professional', 'site coordinators', 'information technology', 'military leaders', 'urgent support', 'supply', 'communications specialist', 'officers', 'inventory specialist', 'outpatient services', 'public assistance', 'resource coordinator', 'response coordinator', 'rapid response team', 'strategy officer', 'pandemic', 'logistics', 'emergency management', 'emergency mgmt', 'emergency technician', 'emergency operations', 'emergency preparedness', 'emergency specialist', 'emergency technician', 'emergency temporary',,

**'public health'**: 'public safety officer', 'program officer', 'fema', 'ep coordinator', 'environmental', 'case investigator', 'coronavirus response fellowship', 'hygienist', 'bio-safety', 'epidemiologist', 'project manager', 'public health', 'program coordinator', 'project officer', 'wash', 'epidemiology', 'epidemiolgist', 'bio-safety',

**'research'**: 'market research', 'editor', 'research fellow', 'policy analyst', 'digital marketing', 'postgraduate', 'editor', 'molecular biology', 'gender equality expert', 'clinical research', 'disease research', 'vaccine',

# Roles -> Broad Categories

"**Frontline**": "nurse", "nursing assistant", "doctor", "allied health", 'Not Specified / Multiple Roles', "support work", "phones/screening", "laboratory science"

"**Coordination**": "administration", "emergency planning", "public health",

"**Decision Support**": "research"
